# 7SL RNA in vertebrate red blood cells

**DOI:** 10.1261/rna.065474.117

**Published:** 2018-07

**Authors:** Gaëlle J.S. Talhouarne, Joseph G. Gall

**Affiliations:** 1 Department of Embryology, Carnegie Institution for Science, Baltimore, Maryland 21218, USA; 2 Department of Biology, Johns Hopkins University, Baltimore, Maryland 21218, USA

**Keywords:** 7SL RNA, noncoding RNA, red blood cells, signal recognition particle

## Abstract

We report that 7SL, the RNA component of the signal recognition particle (SRP), is an abundant noncoding RNA (ncRNA) in mature red blood cells (RBCs) of human, mouse, and the frog *Xenopus.* 7SL RNA in RBCs is not associated with the canonical proteins of the SRP. Instead, it coimmunoprecipitates from a lysate of RBCs with a number of membrane-binding proteins. Human and mouse RBCs also contain a previously undescribed 68 nt RNA, sRN7SL, derived from the “S domain” of 7SL RNA. We discuss the possibility that 7SL RNA is selectively protected from nucleases by association with the RBC membrane. Because 7SL is not associated with the canonical proteins of the SRP, it could represent a nonfunctional remnant of the protein synthetic machinery. Alternatively, it could play a new, as yet undefined role in RBC metabolism.

## INTRODUCTION

In an earlier study we reported the existence of more than 9000 different circular intronic sequences (lariats) in the cytoplasm of the amphibian oocyte (for review, see Talhouarne and Gall 2014). That study was made possible by the ease with which the giant nucleus (germinal vesicle) can be removed manually from the oocyte, leaving a completely pure cytoplasmic fraction for analysis. To determine whether similar intronic sequences occur in other cell types, we examined mammalian red blood cells (RBCs). These cells lose their nuclei during maturation from the erythroblast stage and so provide another convenient source of pure cytoplasm. In studies to be reported elsewhere we find that human and mouse RBCs are, indeed, a rich source of lariat molecules derived from the introns of hundreds of different genes. Surprisingly, we find an even more abundant noncoding RNA (ncRNA) in RBCs, namely 7SL RNA. 7SL RNA is a major component of the eukaryotic signal recognition particle (SRP) (Walter and Blobel 1982), which mediates cotranslational translocation of proteins to the endoplasmic reticulum. RNAs with secondary structures similar to that of 7SL RNA are found in SRPs from all domains of life, such as the 4.5S RNA of bacteria (Struck et al. 1988). 7SL RNA, free of SRP proteins, is also well-known as a component of retrovirus particles (for review, see Telesnitsky and Wolin 2016) and recently has been described in exosomes derived from cultured human fibroblasts (Nabet et al. 2017).

In addition to its abundance, we made two unexpected findings about 7SL RNA in RBCs. First, it is not associated with the canonical SRP proteins, similar to 7SL packaged in viruses, and second, a short 68 nt transcript derived from the 7SL gene also occurs in mammalian RBCs.

## RESULTS AND DISCUSSION

### 7SL RNA is present in red blood cells

**FIGURE 1.**
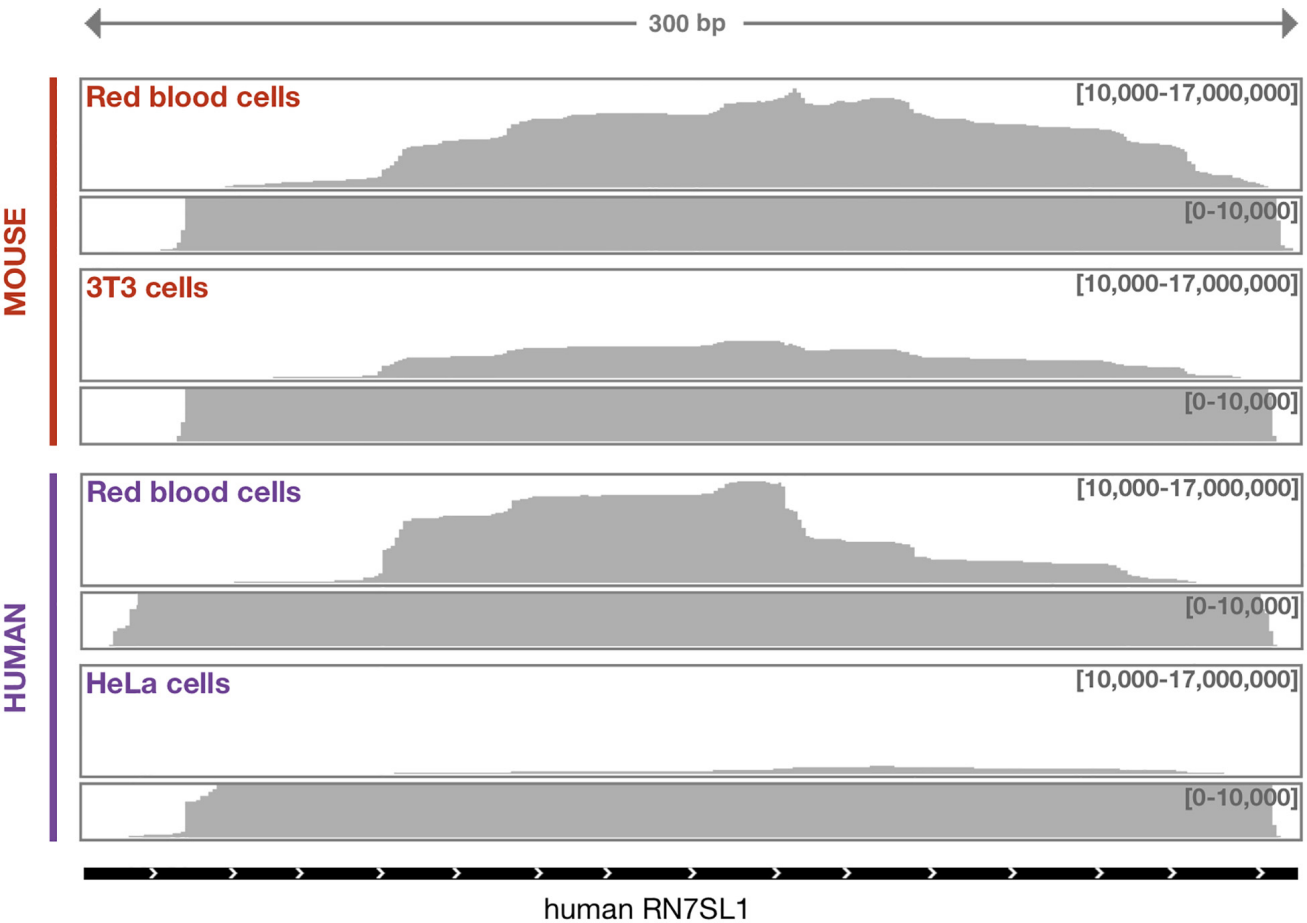
7SL RNA in mouse and human RBCs. Total RNA from cultured cells (mouse 3T3 and human HeLa) and RBCs was sequenced and 45 million reads (100 bp) were mapped against the human RN7SL1 gene and evaluated with the IGV browser (Broad Institute).

Whole blood from mouse or human was centrifuged on a Percoll gradient, which separates the more rapidly sedimenting RBCs from white cells. RNA was purified from the RBCs and subjected to high-throughput sequencing. A large fraction of reads mapped to the RN7SL gene, which encodes 7SL RNA. In the mouse sample, one-quarter of the reads mapped to RN7SL and in the human sample about one-third. Reads mapped across the entire gene sequence, suggesting that RBCs contain full-length 7SL RNA (Fig. 1). In comparable RNA-seq data sets from cultured mouse (3T3) and human cells (HeLa), we found that, respectively, 11% and 7% of reads mapped to RN7SL. In other words, 7SL was three to five times more abundant in RBCs than in cultured cells. In contrast, two other abundant ncRNAs of similar size were less abundant in RBCs than in cultured cells: RNase MRP (nuclear and cytoplasmic) and 7SK (nuclear) were 12 and 350 times less abundant in mouse, and they were four and nine times less abundant in human. Because mammalian RBCs lack nuclei and hence do not transcribe RNA, the enrichment for 7SL RNA in RBCs is probably due to selective retention during RBC maturation.

To confirm the identity of 7SL in our RNA fraction, we gel purified RNA in the range of 200–400 nt and sequenced the ends by RACE. The sequences obtained in this way corresponded to the canonical sequences at the two ends of the 7SL molecule, confirming that full-length 7SL RNA accumulated in RBCs.

**FIGURE 2.**
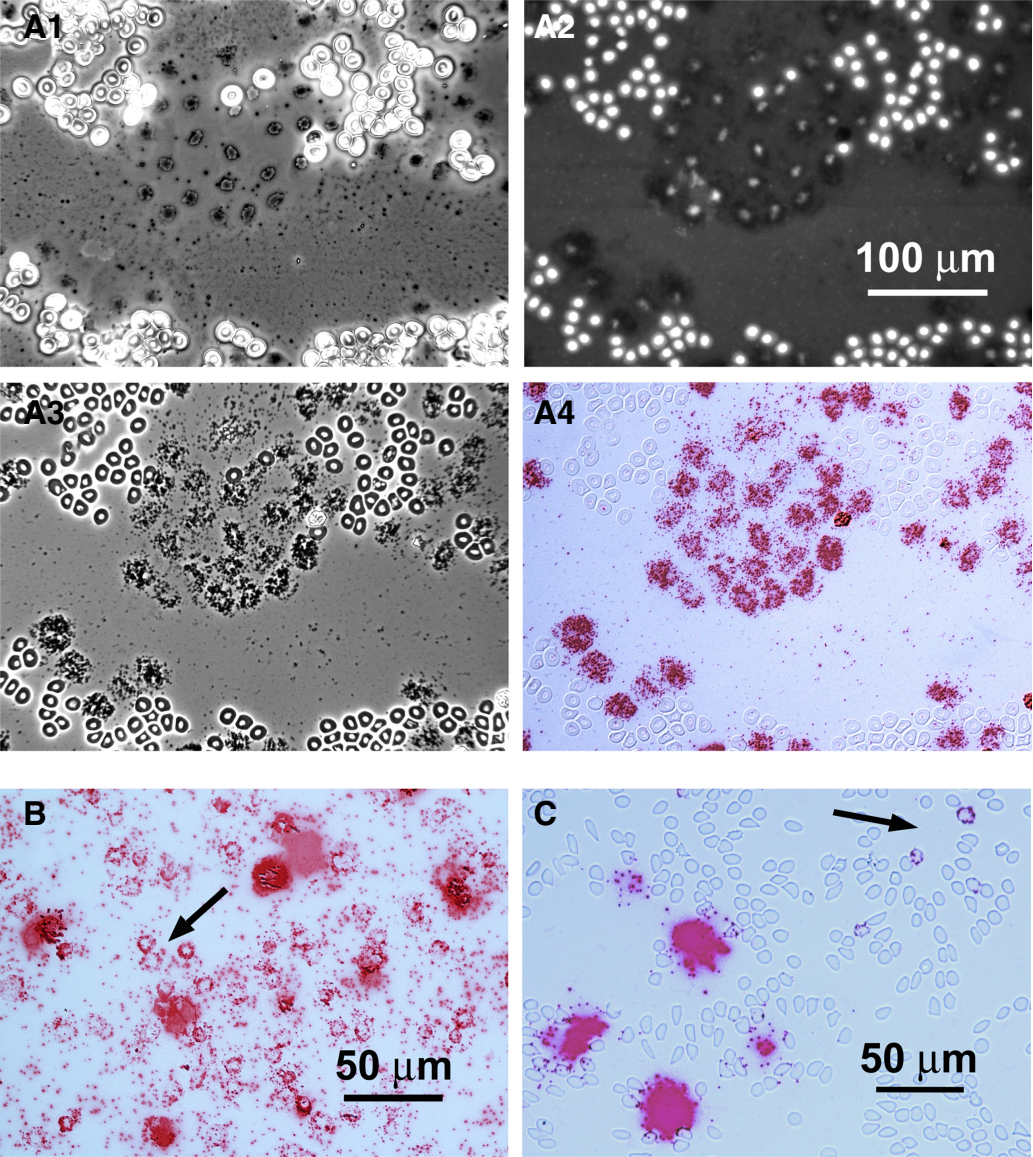
Single molecule in situ hybridization of 7SL RNA. (*A1*–*A4*) The same area of a blood smear of *Xenopus tropicalis*, baked for 1 h at 60^o^, then fixed with ethanol and 4% paraformaldehyde before hybridization. (*A1*) Phase-contrast image taken before in situ hybridization (no mounting medium). A patch of lysed RBCs (black) surrounded by intact RBCs (white). (*A2*–*A4*) The same area after in situ hybridization with a single-molecule probe (red) against 7SL RNA. (*A2*) Fluorescent DAPI stain (white) for DNA. Note that lysed RBCs are each identifiable by their DAPI stain, even though they have lost much of their DNA. The in situ hybridization label does not fluoresce at this wavelength. (*A3*) Phase-contrast image of cells in mounting medium under coverslip. Most RBCs are still intact, showing the nucleus (white) and cytoplasm (black). (*A4*) Bright-field image in which individual red dots presumably represent single 7SL molecules. Note intense label over each lysed RBC but absence of label over intact RBCs. (*B*) A different smear of *X. tropicalis* RBCs, baked 1 h at 60^o^ C but without chemical fixation, then hybridized as in *A*. Some, but not all, intact RBCs (arrow) now show a few dots of label in the cytoplasm (arrow). The label over RBCs that lysed during spreading is so intense that individual molecules are not resolvable. (*C*) Hybridization of human RBCs with the 7SL probe; pretreatment similar to the *X. tropicalis* blood smear in *B*. A very few intact RBCs show label (arrow). The massive patches of label presumably represent single lysed RBCs or clusters of a few lysed RBCs. Because human RBCs lack nuclei, the identification of lysed cells is based on similarity to the condition in *Xenopus* preparations.

To further analyze 7SL RNA in RBCs, we carried out single molecule in situ hybridization on conventional blood smears from *Xenopus*, human, and mouse. We were especially interested to determine whether 7SL RNA was limited to a specific subset of cells, such as immature RBCs, or was present in all mature cells. Because 7SL RNA is only 300 nt long, we used a mixture of three short probes that targeted regions near the middle of the molecule (three “ZZ” BaseScope probes from Advanced Cell Diagnostics). RBCs exhibit a high level of autofluorescence throughout the visible spectrum, requiring the use of a chromogenic detection system (Fast Red dye). We found that RBCs of all three species are remarkably resistant to the hybridization procedure, when prefixed with ethanol and/or paraformaldehyde. Figure 2A1–4 shows a field of RBCs from a blood smear of *X. tropicalis* prefixed with ethanol and 4% paraformaldehyde. Unknown to us before we began these experiments, a small number of RBCs lyse during the spreading procedure. Because *Xenopus* RBCs are nucleated, these lysed cells can still be recognized by the residual DAPI staining of their extruded nuclei (Fig. 2A2). After hybridization, there is no signal in the intact RBCs, but strong hybridization is seen in all cells that lysed during preparation of the smear (Fig. 2A3, A4).

Blood smears that were pretreated by heating to 60°C for 1 h, but not subjected to chemical fixation, showed signal in many intact RBCs (Fig. 2B). The signal was still consistently higher in cells that lysed during the initial spreading procedure. In the case of human and mouse RBCs, which lack nuclei, the position of lysed cells may be inferred from the occasional “blobs” of heavy signal scattered among the intact RBCs (Fig. 2C). The apparent resistance of RBCs to penetration by the probes remains unexplained and is in striking contrast to the ease with which cultured cells or cells in tissue sections can be hybridized. Nevertheless, these in situ hybridization data strongly suggest that many, if not all, mature RBCs of human, mouse, and *Xenopus* contain 7SL RNA, thereby directly confirming the biochemical data.

### A 68 nt fragment of 7SL RNA also occurs in human and mouse RBCs

**FIGURE 3.**
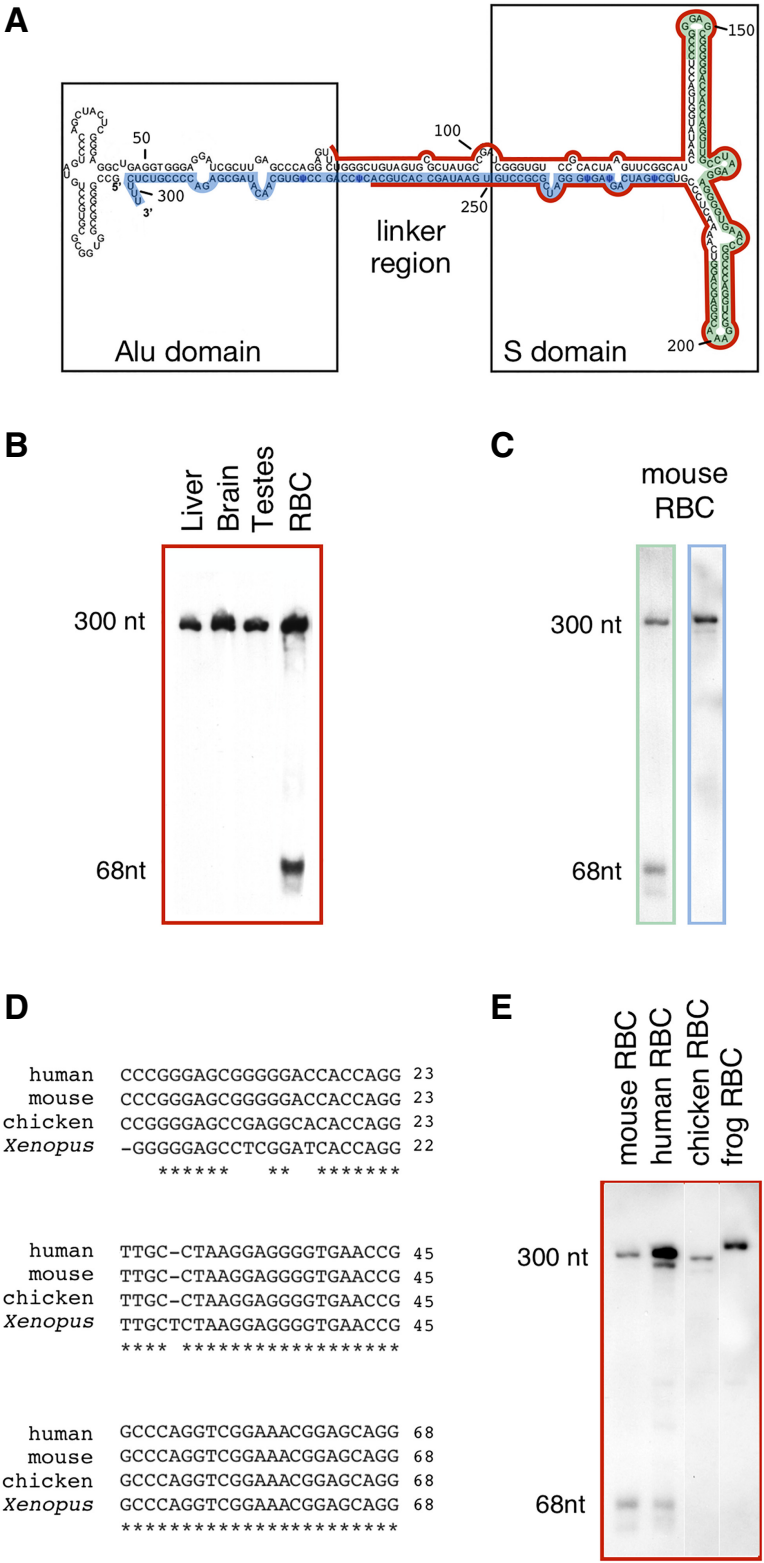
Full-length 7SL RNA and a shorter RNA occur in mammalian RBCs. (*A*) Human 7SL secondary structure: red, green, and blue regions show the probe sequences used for the Northern blots. (*B*) Northern blot analysis of 7SL using the red probe. A small (68-nt) band is detectable only in the RBC sample. Experiment was done twice. (*C*) The region encoding the 68-nt band (sRN7SL) is confirmed by northern blot analysis using as probes the green (*left* lane) and the blue regions (*right* lane). Experiment was done twice. (*D*) Alignment of human, mouse, chicken, and *Xenopus* partial 7SL sequences. Nucleotide positions are relative to human and mouse sRN7SL. (*E*) sRN7SL is detected by Northern blot (red probe) only in mammalian (mouse and human) RBCs (analysis was done on the same membrane). The experiment was done three times.

To further characterize 7SL RNA from blood, we analyzed mouse RBC RNA by northern blotting. The 7SL RNA probe used is shown in red (Fig. 3A), highlighted on the secondary structure of human 7SL RNA (http://rth.dk/resources/rnp/SRPDB/). We detected a band at ∼300 nt, confirming that full-length 7SL RNA is present in RBCs (Fig. 3B). Surprisingly, we detected an additional band that corresponds to a small RNA, presumably derived from 7SL RNA. This small RNA was easily detectable in mouse RBCs, but not in mouse brain, liver, or testis. There are presumably some RBCs in the latter three tissue samples, but at too low a concentration to detect the smaller band.

To determine the sequence of this short RNA, we made a small RNA library from gel-purified 60–80 nt long mouse RBC RNA and cloned it in *E. coli*. Colonies were screened by Southern blotting and positive clones were sequenced. Among the 11 sequenced colonies, eight carried a plasmid containing the sequence between nucleotide 143 and 210 of 7SL (highlighted in green, Fig. 3A). The three other colonies carried a plasmid with a slightly shorter fragment (nucleotides 143–207). We refer to the 68-nt RNA as sRN7SL (short 7SL RNA). To confirm the sequence, we performed Northern blotting with a probe against sRN7SL (green in Fig. 3A) or against a downstream region (blue in Fig. 3A). Full-length 7SL RNA reacted with both probes, but the sRN7SL transcript reacted only with the sRN7SL probe (Fig. 3C).

A 70-nt transcript has been previously annotated at a similar region in the human RN7SL3 gene (ENST00000625125.2). However, this annotation was linked to a predicted piRNA (28 nt), piR-45120 locus DQ577008, identified by Girard et al. (2006). The sequence of sRN7SL overlaps that of the piRNA by only 6 nt. Additionally, the secondary structure predicted for sRN7SL using RNAfold (Lorenz et al. 2011) and mFold (Zuker 2003) differs from that of a pre-miRNA (a hairpin from 60 to 120 nt in length). Therefore, it is unlikely that sRN7SL is a precursor for the piRNA or miRNA.

Ren et al. (2012) proposed that dicer, the protein responsible for miRNA maturation, may cut 7SL RNA into smaller RNAs in cell culture, including a smaller RNA derived from the S-domain. One possibility is that this endonuclease could also be responsible for the accumulation of sRN7SL in RBCs.

Intriguingly, the sRN7SL sequence overlaps a 111-nt 7SL S-domain fragment, namely 7SLrem (for 7SL remnant), found in virus-like particles (Keene et al. 2010). This provides yet another example of a small RNA derived from the S-domain and raises the possibility that the S-domain may carry out multiple functions.

Because the 68 nt region of the RN7SL gene is relatively well conserved across vertebrates (Fig. 3D), we asked whether sRN7SL RNA is expressed in other species. We probed RNA from human, mouse, chicken, and *Xenopus* cell cultures and RBCs with a full-length 7SL RNA probe. We detected full-length 7SL RNA in all samples, but the 68-nt band was present only in the human and mouse RBC samples (Fig. 3E).

### 7SL RNA is associated with multiple proteins in RBCs

The human RBC proteome has been studied extensively by mass spectrometry (for review, see Hegedu˝s et al. 2015), but none of the canonical SRP proteins have been detected, even in the most sensitive assays. Moreover, in an early study Avanesov (1988) fractionated a rabbit reticulocyte extract and identified 7SL RNA in a fraction that contained primarily one protein. This protein was not further characterized, but it had a molecular weight different from that of the SRP proteins. Altogether, the evidence suggests that 7SL RNA in RBCs may be a component of a new RNP.

**FIGURE 4.**
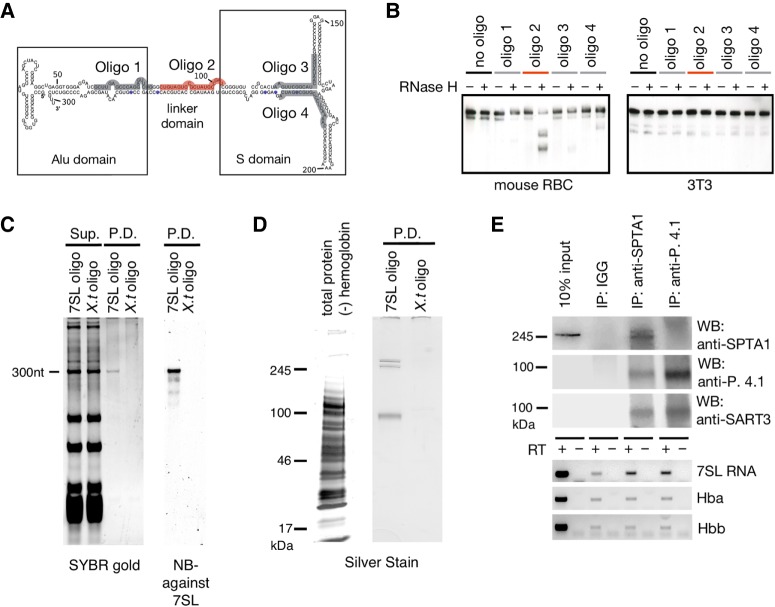
Affinity purification of 7SL RNA. (*A*) Sequence of the four antisense oligonucleotides used to probe 7SL RNA in RBCs. (*B*) 7SL RNA from an RBC lysate was cleaved by RNase H only when it was hybridized to oligo 2 (red). 7SL RNA from mouse 3T3 cells was not cleaved. Experiment was done twice. (*C*) Affinity purification of 7SL. Total RNA in the supernatant (Sup.) and the pull-down (P.D.) after hybridization with a 7SL RNA antisense probe (7SL oligo) or a control probe (*X.t.* oligo). RNA was detected by SYBR gold. Analysis of the pulled-down RNA showed readily detectable 7SL RNA by Northern blotting. Experiment was done twice. (*D*, *left* lane) Total proteins (minus hemoglobins) before pull-down. (*Right* lanes) Proteins in the pull-down fractions shown in *C*. Proteins detected by silver staining. In the 7SL pull-down fraction, the major bands are assumed to be spectrin (α and β chains), protein 4.1 and band3, based on their molecular weights and on the abundance of these proteins in the mass spectroscopic analysis. Experiment was done twice. (*E*) Proteins and RNA pulled down by antibodies against spectrin α and protein 4.1. (*Top* panels) Pulled-down proteins were analyzed by Western blots against spectrin α, protein 4.1 and SART3. SART3 was detected in both pull-downs. Only spectrin alpha was sufficiently abundant to be detected in the input lane. Western blots were performed once. (*Bottom* panels) Pulled-down RNAs were analyzed by RT-PCR using oligos targeting 7SL RNA and two hemoglobin mRNAs, Hba and Hbb. 7SL RNA was detected in both pull-downs. Northern blots were done twice.

To determine what protein(s) might be associated with 7SL RNA in RBCs, we used a strategy based on “capture hybridization analysis of RNA targets” (“CHART”) method (Simon et al. 2011). This method takes advantage of the fact that RNase H specifically cleaves RNA in RNA/DNA hybrid molecules. We used this specificity to predict regions of 7SL RNA that can be targeted for pull-down. We mixed RBC lysate with RNase H and four different DNA oligonucleotides. 7SL RNA in the lysate was efficiently cut by RNase H only in the presence of an oligonucleotide that targeted the linker domain (Fig. 4A). This result suggests that the linker domain may be free of protein and in an open conformation. 7SL RNA from a lysate of mouse 3T3 cells was not cut under the same conditions (Fig. 4B), consistent with 7SL RNA being in different complexes in the two cell types. We used this specific binding to purify the 7SL RNA complex from RBCs.

For the affinity purification of 7SL RNA, we used a “linker targeting” antisense oligo that contained 2′-*O*-methylated nucleotides and a desthiobiotin-conjugated 3′ end nucleotide, similar to oligos used in the CHART method. As a negative control, we used a desthiobiotin-conjugated oligo that is antisense to a *Xenopus*-specific RNA. Pulled-down RNA was run on an acrylamide gel and stained with SYBR Gold. 7SL RNA was detected in the pull-down with the “linker targeting” oligo but not in the control. Moreover, 7SL RNA was the major RNA in the pull-down (Fig. 4C), suggesting that sRN7SL RNA is not directly associated with 7SL RNA. A small sample from the pull-down was also run on a polyacrylamide gel and stained with silver for detection of the most abundant proteins. Three bands were seen (Fig. 4D). Based on the molecular weight of these bands and the results of mass spectrometry analysis (next paragraph), we tentatively identify these bands as the two subunits of spectrin (∼250 and ∼280 kDa) and protein 4.1 and/or band 3 (∼90 kDa).

We analyzed the proteins in the pull-down by mass spectrometry and focused on proteins enriched by at least twofold (Supplemental Fig. S1). Four of the five most abundant proteins in the pull-down were spectrin α and β chains, band 3 and protein 4.1, consistent with the interpretation of the protein gel in Figure 4D. The sensitivity of mass spectrometry allowed us to identify additional proteins, including other actin-associated proteins and many RBC membrane proteins. Of particular interest, we detected one RNA-binding protein: squamous cell carcinoma antigen recognized by T-cells 3 (SART3) (Supplemental Fig. S1), raising the possibility that 7SL might be associated with SART3.

To examine proteins that might be associated with 7SL RNA, we carried-out immunoprecipitations with antibodies against spectrin α and protein 4.1. RBC lysates were depleted of hemoglobin using Ni-NTA agarose beads to lower background noise and subsequently incubated with each of the above antibodies. The antibodies were recovered after 1 h using protein A/G magnetic beads; IGG was used as a negative control. The efficiency of the pull-downs was evaluated by Western blots (Fig. 4E). We tested for 7SL RNA in the pull-downs and found that 7SL co-immunoprecipitated with spectrin α and protein 4.1. Two other abundant RNAs, encoding hemoglobins A and B, were not detected in the pull-downs above background level (Fig. 4E). These data are consistent with 7SL RNA being in a membrane–cytoskeleton complex that contains protein 4.1 and spectrin α.

Because SART3, an RNA-binding protein (Whitmill et al. 2016), was found in the mass spectrometry analysis, we wanted to know whether it also binds 7SL RNA. We tested three commercially available antibodies against SART3, but none of them gave satisfactory immunoprecipitates. At least one, however, did work on Western blots. We found that SART3 coprecipitated with both spectrin α and protein 4.1, when these proteins were immunoprecipated by their cognate antibodies (Fig. 4E). This result suggests that 7SL RNA, SART3, and cytoskeletal proteins are associated in RBCs. We do not know whether SART3 binds 7SL RNA directly. However, SART3 has been shown to bind to tubulin and actin in 293T cells (Timani et al. 2013) and thus it could anchor 7SL RNA to the cytoskeleton of RBCs. Alternatively, because SART3 (Prp24 in yeast) is a recycling factor for U6 snRNA (Bell et al. 2002; Rader and Guthrie 2002), it might be involved in 7SL particle assembly or disassembly. Finally, both SART3 and 7SL RNA have been implicated in p53 mRNA homeostasis (Abdelmohsen et al. 2014; Timani et al. 2015). Therefore, it is possible that they also function together in RBCs.

In summary, we present evidence that 7SL RNA in RBCs is associated with cytoskeletal proteins, including protein 4.1 and spectrin α. SART3 is also associated with these cytoskeletal proteins, although we have not demonstrated direct binding between SART3 and 7SL RNA. The functional significance of these associations remains to be determined.

## MATERIALS AND METHODS

### RBC purification

Blood samples were centrifuged at 300*g* for 10 min at room temperature to pellet the cells. The supernatant plasma was removed and the cells were resuspended in 1× PBS. The suspension was layered onto a Percoll gradient (50%–80%) and centrifuged at 1000*g* for 30 min at room temperature. RBCs accumulated near the bottom of the tube and were collected free of white blood cells. Before use, they were washed three times in 1× PBS.

### Blood smears

A drop of whole blood or red blood cells was spread quickly on a microscope slide and dried overnight or longer. In some cases the smear was fixed for up to 1 h in 100% ethanol and/or 4% paraformaldehyde.

### RNA purification

RNA was extracted with TRIzol reagent (Ambion) and purified with the Direct-zol RNA MiniPrep kit (Zymo Research). DNase I treatment was performed on the Direct-zol column. RNA concentration was determined with a Nanodrop apparatus (Thermo Fisher Scientific) and its integrity (size distribution) with a Bioanalyzer 2100 (Agilent).

### Single molecule in situ hybridization

The probe consisted of three “ZZ” oligonucleotide pairs from Advanced Cell Diagnostics, which are complementary to the following three regions of human 7SL:

bases 99–138: CGATCGGGTGTCCGCACTAAGTTCGGCATCAATATGGTG,
bases 165–199: GTTGCCTAAGGAGGGGTGAACCGGCCCAGGTCGGA, and
bases 200–242: AACGGAGCAGGTCAAAACTCCCGTGCTGATCAGTAGTGGGATC.

The “ZZ” oligonucleotide pairs are attached to a proprietary amplification target, which in turn is detected by a proprietary set of oligonucleotides. The latter are finally detected by Fast Red dye. Each red “dot” in the final preparation presumably represents a single RNA molecule.

### Northern blots

Up to 1 µg of RNA per sample was separated on an 8% polyacrylamide/8 M urea gel and transferred onto a nylon membrane (Zeta Probe GT, Bio-Rad). RNA was probed with dsDNA labeled with digoxigenin (DIG)-dUTP in hybridization buffer (Roche) and detected using an anti-DIG antibody conjugated with alkaline phosphatase (REF 11207733910**,** Roche) and CDP-*Star* chemiluminescent substrate (Roche).

### RNase H

Cells were homogenized in 500 µL RNase H buffer: 50 mM HEPES pH 7.5, 150 mM NaCl, 5 mM MgCl_2_, 1 mM DTT, 0.2 U RNasin (Promega), one protease inhibitor cocktail tablet (cOmplete Tablets–Mini EASYpack, Roche) and 1% Tween-20. Twenty microliters of cleared lysate was incubated 1 h at 37°C with 5 units of RNase H and 1 mM antisense oligo prior to RNA purification.

### RACE/small RNA library

RNA was run on an 8% polyacrylamide–8 M urea gel. RNAs ranging from 200–400 nt and 50–100 nt were cut from the gel and extracted overnight with 300 mM NaOAc, 1mM EDTA, 0.25% SDS at room temperature. Oligos were ligated to the 5′ and 3′ end according to the NEBNext Multiplex Small RNA Library Prep protocol. The RNA was converted to cDNA with an oligo that targeted the ligated sequence at the 3′ end. It was then amplified either with NEBNext oligos to make a library or with internal oligos to sequence the end of 7SL RNA specifically. All products were analyzed by TA cloning.

### RNA pull-down assay for mass spectrometry

One hundred microliter of packed mouse red blood cells were homogenized in lysis buffer (20 mM Tris at pH 7.5, 150 mM NaCl, 5 mM MgCl_2_, 1 mM DTT, 0.2 unit of RNasin [Promega], one protease inhibitor cocktail tablet [cOmplete Tablets–Mini EASYpack, Roche], and 1% Triton X-100) and incubated 1 h at 37°C with 5 µL of 10 µM biotinylated antisense oligonucleotide purchased from Integrated DNA Technologies. The sequence of the antisense oligonucleotide to mouse 7SL was /5BioTinTEG/iSp18/mAmUmCmGmGmCmAmUmAmGmCmGmCmAmCmUmAmCmAmGmCmCmCmAmGmAmAmCmUmCmCmUmGmGmAmCmUmCmAmAmG/iSp18/3BioTEG/. The sequence of the antisense oligonucleotides to *Xenopus tropicalis* faf2 intron 2 was mGmAmUmUmUmGmAmCmCmAmCmAmCmAmCmAmCmAmCmAmGmUmU/iSp18/3BioTEG/ and mGmUmAmUmUmUmGmUmAmUmGmCmUmCmAmGmAmCmCmUmGmC/iSp18/3BioTEG/.

One-hundred microliters of washed Dynabeads MyOne Streptavidin T1 (Thermo Fisher Scientific) was added, and the mix was further incubated at 37°C for 1 h. Beads were washed in 1 mL of lysis buffer five times for 10 min. RNA was purified from beads that were directly added to TRIzol (10%), run on an 8% acrylamide/8 M urea gel, and analyzed by SYBR Gold and Northern blotting. The pulled-down complexes were eluted with 50 µL of nanopure water by heating gradually (0.5°C/sec) to 70°C (based on Holmberg et al. 2005). Five microliters of the eluate was run on a 4%–15% polyacrylamide gel (Mini-PROTEAN TGX precast protein gels from Bio-Rad) and silver-stained (Pierce Silver Stain for Mass Spectrometry from Thermo Fisher Scientific) according to the manufacturer's protocol.

Mass spectrometry was carried out at the Johns Hopkins Medical School Mass Spectrometry and Proteomics Facility. Pulled-down complexes were digested with trypsin, desalted, and analyzed by liquid chromatography tandem-mass spectrometry on a Q Exactive Plus instrument (Thermo Fisher Scientific). The output was searched against refseq2015_mus_musculus database with PD1.4-Mascot.

### Immunoprecipitation

Mouse red blood cells were homogenized in lysis buffer (20 mM Tris at pH 7.5, 150 mM NaCl, 5 mM MgCl_2_, 1 mM DTT, 0.2 U RNasin [Promega], one protease inhibitor cocktail tablet [cOmplete Tablets–Mini EASYpack, Roche], and 1% Triton-X100). After centrifugation to clear the lysate, hemoglobin was depleted using 1 volume of NI-NTA agarose beads (Qiagen) for 1 volume of lysate (Ringrose et al. 2008). Five-hundred-microliter aliquots of lysate were incubated 1 h at 37°C with 1 µL of anti-spectrin (ABclonal, catalog no: A12355), 5 µL of anti-protein 4.1 (Proteintech, catalog no. 13014–1-AP), or 5 µL of IGG (kindly provided by the Bortvin laboratory, Carnegie Institution) and incubated an additional 1 h at 37°C with protein A/G magnetic beads (Pierce). Beads were washed three times for 15 min at 37°C before the RNA was eluted with TRIzol and the protein with reducing sample buffer. Pulled-down proteins were analyzed by Western blot. Primary antibodies were diluted (in 5% milk) 1:3000 for rabbit anti-spectrin and 1:1000 for rabbit anti-protein 4.1 and rabbit anti-sart3 (Proteintech, catalog no. 18025-1-AP). HRP-conjugated goat anti-rabbit antibody was used at 1:15,000. Detection was with SuperSignal West Dura Extended Duration Substrate (Thermo Fisher Scientific) and monitored with a LI-COR Odyssey Fc Imager. Pulled-down RNA was analyzed using OneStep RT-PCR (Qiagen), according to the manufacturer's protocol, and the following oligos:

7SL oligo: TCTGGGCTGTAGTGCGCTAT and GGCTGGAGTGCAGTGGCTAT,
Hba oligo: CCAAGACCTACTTCCCTCACTT and GAAGGCAGCTTAACGGTACTTG, and
Hbb oligo: CGTTTGCTTCTGATTCTGTTGT and GGCAGAGGATAGGTCTCCAAA.

### Library preparation, sequencing, and sequence analysis

Libraries were prepared using TrueSeq stranded total RNA sample preparation (Illumina). Sequencing was performed on an Illumina HiSeq 2000 sequencer with 100 bp single-end reads. Reads were aligned with TopHat (v2.0.7) to the mouse genome (v10) or the human genome (v19). 7SL and RNase MRP reads were quantified using Bedtools (v2.15.0).

## SUPPLEMENTAL MATERIAL

Supplemental material is available for this article.

## Supplementary Material

Supplemental Material
