# Supplementary material for: 7SL RNA in vertebrate red blood cells
Source: RNA. 2018 Jul;24(7):908–14. doi: 10.1261/rna.065474.117 (PMC6004055; doi:10.1261/rna.065474.117)
Supplement: Supplemental Material [file supp_24_7_908__index.html]

7SL RNA in Vertebrate Red Blood Cells — 7SL RNA in vertebrate red blood cells — Supplemental Material 

# 7SL RNA in vertebrate red blood cells

## Supplemental Material

- Supplemental\_Figure\_S1\_Legend.docx
- Supplemental\_Figure\_S1.tiff
