# Supplementary material for: 7SL RNA in vertebrate red blood cells
Source: RNA. 2018 Jul;24(7):908–14. doi: 10.1261/rna.065474.117 (PMC6004055; doi:10.1261/rna.065474.117)
Supplement: Supplemental Material [file supp_065474.117_Supplemental_Figure_S1_Legend.docx]

**Figure S1. Detection of proteins by mass spectrometry.** The list contains proteins that had at least 20 peptides detected and were enriched by at least 2-fold in a sample pulled-down by an antisense oligo against 7SL RNA, relative to those pulled-down by a control antisense oligo against *X. tropicalis* faf2 intron2 (*X.t* oligo). Cytoskeletal proteins are marked in green, hemoglobins in red, and plasma membrane proteins in blue. Note that none of the canonical proteins of the SRP particle were detected.
