# Supplementary figures and images for: 7SL RNA in vertebrate red blood cells
Source: RNA. 2018 Jul;24(7):908–14. doi: 10.1261/rna.065474.117 (PMC6004055; doi:10.1261/rna.065474.117)

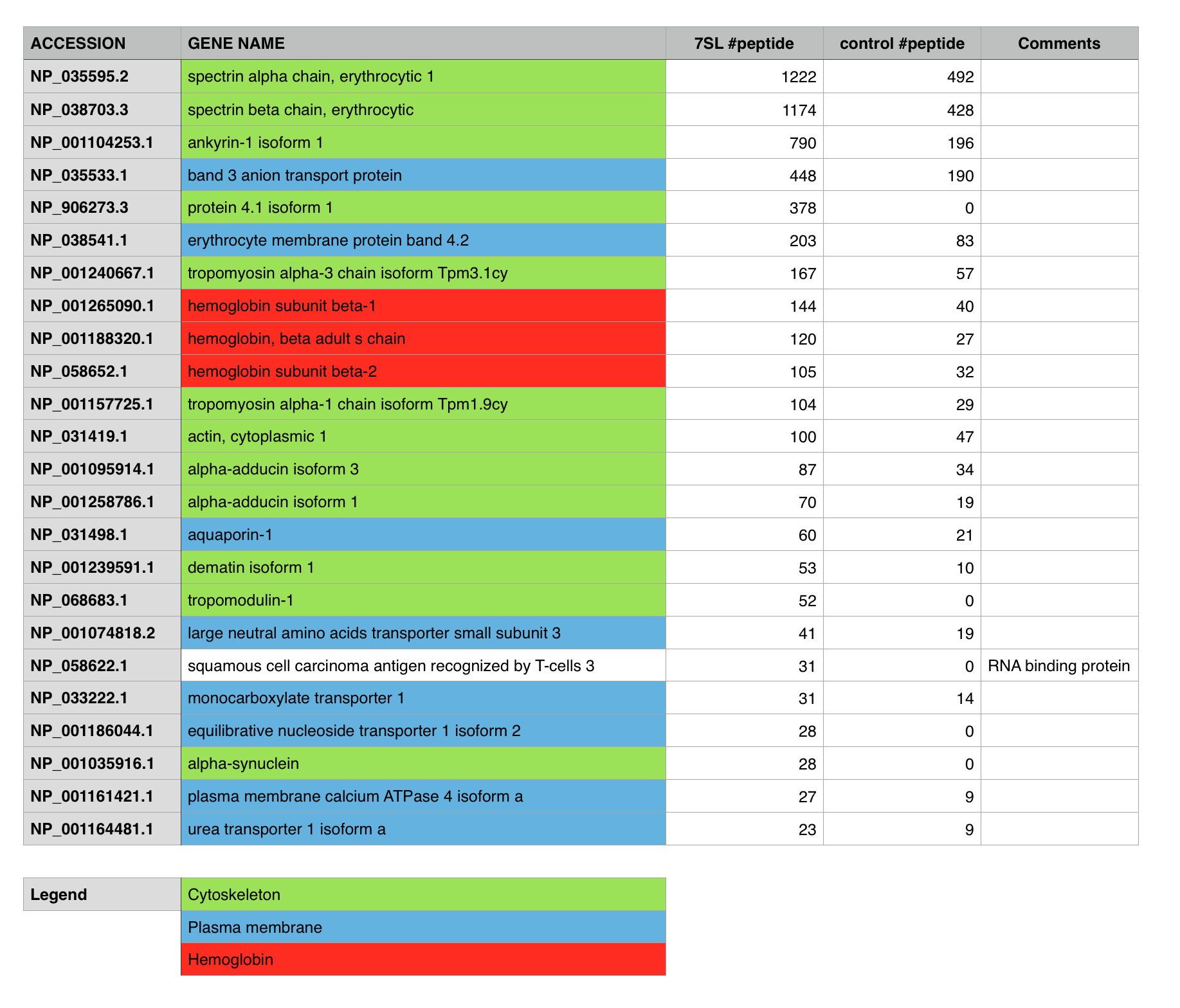

Supplement: Supplemental Material [file supp_065474.117_Supplemental_Figure_S1.tif]
